# Association between Second-Hand Exposure to E-Cigarettes at Home and Exacerbations in Children with Asthma

**DOI:** 10.3390/children11030356

**Published:** 2024-03-18

**Authors:** Serena Costantino, Arianna Torre, Simone Foti Randazzese, Salvatore Antonio Mollica, Federico Motta, Domenico Busceti, Federica Ferrante, Lucia Caminiti, Giuseppe Crisafulli, Sara Manti

**Affiliations:** Pediatric Unit, Department of Human Pathology in Adult and Developmental Age “Gaetano Barresi”, University of Messina, Via Consolare Valeria, 1, 98124 Messina, Italy; serena.costantino@studenti.unime.it (S.C.); trrrnn95p56f158l@studenti.unime.it (A.T.); salvatore.mollica@studenti.unime.it (S.A.M.); f-motta@hotmail.it (F.M.); nicobusceti@virgilio.it (D.B.); frrfrc88m58c351g@studenti.unime.it (F.F.); lucia.caminiti@polime.it (L.C.); crisafulli.giuseppe@unime.it (G.C.); sara.manti@unime.it (S.M.)

**Keywords:** asthma, children, e-cigarettes, second-hand exposure

## Abstract

Several studies have shown the effects of e-cigarettes in adults. Nowadays, few data are available in the pediatric population. This study aims to assess the relationship between asthma exacerbations and home exposure to e-cigarettes. We conducted a pilot, retrospective, monocenter, observational study. Demographic and clinical data were collected, including number of asthma exacerbations, need for rescue therapy and/or therapeutic step-up, and Asthma Control Test (ACT) and children-Asthma Control Test (c-ACT) scores. The cohort consisted of 54 patients (5–17 years old), divided into two groups: A, including patients exposed to e-cigarette aerosols; B, including unexposed patients. The statistical analysis showed no relevant variation in the number of asthma symptomatic days and need for rescue therapy in group A versus group B (*p* = 0.27 and 0.19, respectively). There were no statistically significant variations when also considering the number of patients who needed a therapeutic step-up (*p* = 0.3). The mean values of ACT and c-ACT were, respectively, 17.2 ± 7.6 and 18.3 ± 5.6 in group A and 19.6 ± 3.8 and 14.6 ± 5.8 in group B (*p* = 0.3 and 0.4, respectively). Although we did not find a statistically significant correlation between second-hand e-cigarette exposure and asthma exacerbations, our findings suggest that asthmatic children exposed second-hand to e-cigarettes may have increased risk of asthma symptomatic days. Future research is warranted.

## 1. Introduction

Asthma is the most common airway disease in the pediatric age group, characterized by chronic inflammation and airway hyper-reactivity leading to coughing, wheezing, difficulty breathing, and chest tightness [1]. It is estimated that more than 300 million people worldwide are affected by asthma [2]. In total, 5% of asthmatic patients experience a severe phenotype characterized by frequent exacerbations, need for hospitalization, complications, and poor quality of life (QoL) with a significant psychosocial impact [3,4]. Implementing preventive measures, such as patient education programs, awareness campaigns, and improved access to asthma management resources, may help to reduce the burden of asthma exacerbations [5]. Environmental tobacco is universally reported as a risk factor for exacerbations in asthmatic subjects [6,7]. It is well known that tobacco smoking represents the most common preventable cause of mortality and morbidity in the world [8]. The reduction of its prevalence both in the pediatric and adult population is one of the most outstanding achievements in public health [9]. However, especially among adolescents, the use of electronic cigarettes (e-cigarettes) has spread rapidly due to the development of new products, advertising, promotion, and marketing strategies that suggest vaping as a risk-free solution [10]. It is particularly worrying that the number of users continues to grow, with an estimated 80 million users worldwide by 2023 [11]. Electronic nicotine delivery systems (ENDSs) are commonly considered safer than traditional combustible cigarettes (CCs) [12]. However, it is still known that vaping represents an independent risk factor, even in healthy subjects that are not habitual smokers, for the development of respiratory symptoms such as bronchoconstriction and cough [13]. Several studies involving the adult population have shown the short- and long-term effects on respiratory health caused by e-cigarettes, even in cases of passive or second-hand exposure (SHE) to second-hand aerosol (SHA) [14]. The relationship between passive exposure to e-cigarette aerosols and an increased risk of developing respiratory symptoms such as dyspnea and bronchitis was recently proved [15]. Establishing evidence of adverse health effects caused by second-hand nicotine vaping exposure could represent a valid motivation for minimizing household exposure and imposing restrictions on vaping in public spaces. While exposure to particulate from second-hand e-cigarettes is lower than that caused by CCs [16], the concentration of ultrafine particles in e-cigarette aerosols may exceed those in cigarette smoke, with the toxins transported to the distal airways and alveoli [17]. E-cigarette aerosol also includes volatile aldehydes and oxidant metals that may induce lung toxicity [17,18,19,20,21,22]. Nowadays, the presence of toxic compounds in the indoor air of e-cigarette smokers’ houses, such as particulate matter (PM) 2,5 and PM 10, nicotine, and volatile organic compounds (VOCs), has been demonstrated [23]. Therefore, the use of ENDSs indoors should be absolutely avoided, especially in the presence of children and adolescents. Despite emerging evidence linking e-cigarette use to negative health outcomes, there is limited exploration of the health effects associated with exposure to second-hand nicotine vaping [24]. A recent cross-sectional study revealed a connection between second-hand nicotine vaping exposure and asthma exacerbations [25]. Nevertheless, to date, few data are available on the pediatric population. A few studies have explored the health consequences of exposure to SHA [25,26,27,28,29,30,31], revealing elevated urinary cotinine levels, changes in respiratory mechanics, decreased fractional exhaled nitric oxide (FeNO), and a correlation with asthma exacerbations in adolescents [25,26,27]. So far, only one study in the literature shows an association between exposure to passive smoke from e-cigarettes containing nicotine and an increased risk of developing respiratory symptoms such as wheezing or bronchitis [32]. With the aim to fill this gap, we retrospectively investigated the correlation between asthma symptoms and exposure to second-hand nicotine vaping in a pediatric population diagnosed with asthma.

## 2. Materials and Methods

### 2.1. Type of Study

A retrospective, monocenter, observational study was conducted from 1 January to 1 May 2023 at the Pediatric Unit, “Gaetano Martino” Hospital, University of Messina, Italy. At the outpatient visit, demographic and clinical data, including age, gender, race, comorbidities, socioeconomic status, education level of patients and caregivers, exposure to e-cigarette aerosols (yes/no), number of smoked e-cigarettes in the last year, number of asthma exacerbations in the previous year, and need for rescue therapy and/or therapeutic step-up were collected. A study-specific questionnaire was administered to all participants. Data were extracted and included in the final analysis. The use by parents of e-cigarettes, both in terms of never having used them and current use, was determined. Participants were classified as “unexposed” if their parents reported never using e-cigarettes and “exposed” if their parents reported using e-cigarettes for at least one day in the last year. Furthermore, the frequency of exposure to SHA within the household in the last year was evaluated by asking parents about their daily consumption of e-cigarettes/ENDSs with the following questions: “How many e-cigarettes/ENDS do you smoke per day?” and “How many current users of e-cigarette/ENDS live in your home?”. Asthma symptoms were defined according to the current guidelines [33]. Asthma Control Test (ACT) and children-Asthma Control Test (c-ACT) scores were also administered at the time of the visit. In this regard, each patient was required to fill a diary of symptoms in which to report the number of symptomatic days, severity of symptoms, need for treatment, and triggers (such as infection, exposure to pollution, and physical activity) that could contribute to the reported flare up. Compliance with treatment and inhalation techniques was also assessed during each visit. A personalized, stepwise action plan with brief education to improve the patient’s ability to recognize and self-start treatment for worsening asthma symptoms was provided to each participant in addition to usual care and post-discharge after an acute exacerbation.

### 2.2. Subjects and Eligibility Criteria

Pediatric patients of both genders aged 5–17 years old affected by asthma were recruited. The following inclusion criteria were adopted: diagnosis of asthma and exposure to aerosol from e-cigarettes. Diagnosis of asthma was based on the following: symptoms or features supporting the diagnosis of asthma, positive bronchodilator responsiveness test (increase in forced expiratory volume in the 1st second, FEV1, from baseline of 12% predicted), excessive variability in twice-daily peak expiratory flow (PEF) over 2 weeks, positive exercise challenge test, excessive variation in lung function between outpatient visits, and documented expiratory airflow limitation [33].

Asthmatic patients exposed to aerosols from CCs were excluded.

### 2.3. Data Analysis

The data were analyzed using Microsoft Excel version 2023 and Statistical Package for Social Sciences (SPSS) version 22.0. Data were considered statistically significant with a *p* value < 0.05. The continuous variables were analyzed using descriptive statistics and expressed as mean ± standard deviation (SD). The ordinary variables were expressed as percentages. Fisher’s test or the Pearson chi-squared test (Pearson coefficient of correlation) and the independent t-test were used for qualitative and continuous variables, respectively.

### 2.4. Ethics

Study participants and their caregivers provided informed consent as part of the study protocol. This study was conducted according to Good Clinical Practice and in compliance with the Declaration of Helsinki with successive amendments. As this study used anonymized and unidentifiable data routinely collected, ethical committee approval was not required.

## 3. Results

A total of 54 children with asthma were included in this study. Specifically, 39 children with intermittent asthma, 9 with moderate asthma, and 6 with severe asthma were recruited, with a mean duration of the disease of 3.4 ± 3.1 years. In total, 35/54 (65%) were 5–11 years old, and 19/54 (35%) were 12–17 years old. Based on e-cigarette aerosols exposure, the enrolled population was stratified into two groups: group A, including 27 exposed patients; and group B, including 27 unexposed patients. The demographic and clinical findings of the enrolled population are shown in Table 1.

The mean age was 9.7 ± 3.6 years for the exposed group and 11.8 ± 4.1 for the unexposed group.

Regarding group A: 16/27 (59.3%) were male, and 11/27 (40.7%) were female; 26/27 (96.3%) were Caucasian, and 1/27 (3.7%) was Asian; 17/27 (63%) resided in the city, and 10/27 (37%) lived in the countryside; 18/27 (66.7%) presented allergic rhinitis (AR); 1/27 (3.7%) was treated with omalizumab.

Regarding group B: 16/27 (59.3%) were male, and 11/27 (40.7%) were female; 25/27 (92.6%) were Caucasian, 1/27 (3.7%) was Asian, and 1/27 (3.7%) was African; 15/27 (55.6%) resided in the city, and 12/27 (44.4%) lived in the countryside; 22/27 (81.5%) presented allergic rhinitis (AR) and 2/27 (7.4%) food allergy (FA); 1/27 (3.7%) was treated with omalizumab.

The results are reported in Table 2.

The analysis of the data collected revealed that the number of asthma exacerbations in the previous year was higher in group A (208 vs. 155, *p* = 0.27). In our study, specifically, we considered an asthma exacerbation to be every time a medical diagnosis was reported by caregivers. All patients had good compliance with treatment and followed our indications regarding the correct inhaler technique including the use of a spacer. Concerning the treatment, at the time of enrollment (T0) all the patients used inhaled corticosteroids (ICSs) according to step 1 or 2 of the Global Initiative for Asthma (GINA), except for the two patients treated with omalizumab. The patients in group A more frequently needed rescue therapy (six times vs. once, *p* = 0.19). Furthmore, 44.4% of patients in group A required a therapeutic step-up vs. 29.6 of group B. In this case, the final treatment included all the medications indicated in step 3 of GINA. We considered the possibility of a therapeutic step-up in case asthma control was not achieved, after verifying that the prescribed medications were used property and consistently administrated with proper technique and with adequate adherence. Regarding asthma control, the mean values of ACT and c-ACT were, respectively, 17.2 ± 7.6 and 18.3 ± 5.6 in group A and 19.6 ± 3.8 and 14.6 ± 5.8 in group B (*p* = 0.3 and *p* = 0.4). All the results were not statistically significant.

## 4. Discussion

The aim of this study was to demonstrate the existing relationship between passive exposure to e-cigarette aerosols and asthma exacerbations in a population of children with diagnosed asthma living in the south of Italy. Specifically, we aimed to prove that children whose parents were e-cigarette smokers were more prone to asthma exacerbations than those belonging to the control group, and even though the *p*-values obtained did not let us reach the desired statistical power, our data seem to suggest an apparent connection between these two factors. Moreover, our results clearly showed that patients exposed to second-hand e-cigarette smoking needed a more aggressive treatment in terms of frequency of rescue therapy requirement and necessity of therapeutic step-up, although data coming from the evaluation of ACT and c-ACT scores did not demonstrate a significant difference as regards asthma control between the two groups. Even though our overall sample size was not large enough to make our results statistically meaningful, the current study explicitly identifies the harmful role of e-aerosol on the respiratory tract. The age difference between the two groups seems to support the traditional hypothesis that younger age correlates with an increased frequency of asthma exacerbations as a result of an underdeveloped immune system, smaller-diameter airways, and an increased sensitivity to respiratory infections. However, this simplistic view fails to consider the multifactorial nature of asthma exacerbations and overlooks the potential influence of other variables that may contribute to the risk of exacerbation among pediatric asthma patients. Although these findings call into question the simplistic idea that only the youngest age predisposes children to more frequent asthmatic exacerbations, our findings highlight the importance of considering a full range of factors in assessing the risk of exacerbation among patients with pediatric asthma. By addressing modifiable risk factors such as allergen exposure and drug adherence, healthcare professionals can potentially reduce exacerbation rates and improve asthma outcomes in all age groups in children. SHA is a kind of environmental pollution produced by ENDSs which contains PM, nicotine, 1,2 propanediol, VOCs such as acrolein, benzene, toluene, formaldehyde, and acetaldehyde, polycyclic aromatic hydrocarbons, and heavy metals [34]. Although the exposure to PM generated by e-cigarettes as second-hand smoke seems less than that from traditional cigarettes [28], it is worth noting that the ultrafine particles present in e-cigarette aerosols could potentially exceed those found in cigarette smoke. This raises concerns about potentially transporting toxins to the distal airways and alveoli [35]. SHA exposure’s potential risks are not only related to the substances contained but also to the possibility that the heating of the components of e-liquid could cause the release of a significant amount of acetaldehyde and formaldehyde or carbonyls, which are well known for their toxic, irritant, and potentially cancerogenic effects on the airways when inhaled [36]. In particular, the existing relationship between exposure to formaldehyde and asthma exacerbations has been reported in a study by Arts et al. [37] in which this substance is described as an irritant agent able to provoke irritant-induced asthmatic reactions in non-allergic individuals as well. Despite a growing body of evidence linking e-cigarette use to adverse health outcomes, there has been a limited exploration of the health effects associated with exposure to second-hand nicotine vaping in the pediatric population [25]. Recently, Tackett et al. conducted a study with the aim to evaluate the actual incidence of children’s exposure to second-hand nicotine and the perception of harm of their parents as regards cigarette and e-cigarette smoke. They divided caregivers into three groups: exclusive cigarette users (*n* = 19), exclusive e-cigarette users (*n* = 12), and nonusers (*n* = 20), and asked them to complete self-report questionnaires regarding their perception of harm; their children’s nicotine exposure was determined through the analysis of urinary cotinine. It was found that e-cigarette users considered these products less harmful than the other two groups, and, additionally, exposure to nicotine seemed to be similar in children whose parents were cigarette and e-cigarette users [38]. Moreover, recent data from the 2018 Minnesota Adult Tobacco Survey, which investigated Minnesota residents’ habits regarding the use of tobacco products (including e-cigarettes), indicated that only 29% of adults using e-cigarettes and living with children prohibited vaping at home vs. 82% of smokers with children who maintained smoke-free homes [39]; so, parents might hold lower risk perceptions regarding children’s exposure to second-hand vaping at home compared to their beliefs on second-hand tobacco smoke. Ward et al. collected data from 28 qualitative interviews regarding e-cigarette users’ views, including the topic of “vaping around children”. The participants were classified by their demographic characteristics according to gender (50% female), ethnicity (96.4% White British), professional occupation, age, and on the basis of having children under 18 years old. Fourteen of them lived with a child under the age of 18. They were also classified as “recreational vapers” and “medicinal vapers”, the latter being ones who used e-cigarettes in order to replace or reduce smoking for health improvement. The positions of parents belonging to these two different groups were quite different as concerns the issue of protecting their children from intergenerational transmission of vaping; in particular, those parents who vaped for medicinal reasons made efforts to limit their vaping in the presence of their children to avoid setting a vaping example. On the other hand, parents who vaped for recreational purposes considered e-cigarette use a responsible choice that might protect their children from SHE to cigarette smoke [40]. Nam et al. conducted an exploratory study interviewing 15 subjects on the topic of e-cigarette risk perceptions and parental role and investigated how both of these factors had an impact on vaping behaviors at home. The participants satisfied two inclusion criteria: they lived with a child aged 5–14 and had used e-cigarettes at least 2 days per week in the previous 4 weeks. People who used other types of tobacco products were excluded. The interviews were conducted and recorded virtually, were guided in order to obtain both objective and subjective information, and lasted until theoretical and thematic saturation was reached. The sample (15 participants) was mainly composed of black (11) males (13), and their ages ranged from 24 to 41. Most of them believed that e-cigarette use was not as harmful as that of traditional cigarettes; so, what emerged during these interviews was the need for future health campaigns that could not only increase knowledge about the risks related to second-hand e-cigarettes exposure but also focus on the caregiver role and the importance of productive communication with their children on this controversial issue [41]. Bayly et al., in their school-based cross-sectional survey enrolling 33,558 high school students and 36,082 middle school students (aged 11–17 years) with a self-reported diagnosis of asthma (*n* = 11,830), wanted to evaluate the possible existing relationship between SHA exposure and asthma exacerbations. Individuals who had never received an asthma diagnosis were excluded from the study. Participants were stratified according to demographic features, according to their eventual status as tobacco users (cigarettes, cigars, hookah, and ENDSs), and according to their exposure to second-hand smoke and second-hand ENDS aerosols. The results showed that 21% of the enrolled patients reported having an asthma attack in the past 12 months, and 33% of them reported second-hand ENDS aerosol exposure. The latter was also associated with a higher risk of asthma attacks in the previous 12 months [26]. The study by Islam et al., performed with the purpose of analyzing the association between SHA exposure and adverse respiratory symptoms among young adults, came to similar conclusions. Their investigation included 2097 children and adolescents enrolled from schools throughout Southern California. Data were obtained from repeated annual surveys that participants had to fill out from 2014 to 2019. Participants reported no primary vaping or smoking in the previous 30 days and no diagnosis of asthma. The authors reported a significant association between exposure to passive smoke from second-hand exposure to e-cigarettes and risk of developing respiratory symptoms such as bronchitic symptoms or shortness of breath, even though the higher rates of documented wheezing were not statistically meaningful [32]. Considering the above-mentioned results, our study highlights the importance of launching health campaigns to educate children, adolescents, and their families about the harmful effects of both direct and indirect exposure to e-cigarette aerosols. It also advocates for supporting health policy initiatives that restrict e-cigarette use across all age groups. Our work, however, has some limitations, including a small sample size. Furthermore, despite using the standardized ACT or c-ACT questionnaire, the reliance on self-reported data introduces the possibility of information bias that, together with the absence of details regarding participants’ adherence to asthma treatment, represents other constraints of our study. Since our study was retrospective, we were not able to record simultaneously to asthma exacerbations an objective parameter that could undoubtedly identify e-cigarette smoking as the only cause of the relapses; plus, we did not take into account other triggers that could have had an effect on the respiratory system, but we believe that all of these factors, although undeniable limits of the present work, do not represent important confounding factors so far as they could have affected both of the populations under study. Additionally, our research is limited by the need for more data on the magnitude of the exposure to second-hand aerosols, not considering the duration or the degree of environmental pollution. Lastly, we cannot exclude the assumptions of those whose parents use e-cigarettes at home/in front of their children, which may or may not be accurate.

## 5. Conclusions

According to the current literature, our data highlight the importance of the prevention of the vaping epidemic and passive exposure to e-cigarettes, even among children and adolescents. Implementing educational programs to increase awareness about the risks of vaping among children and emphasizing the potential impact on respiratory health, especially for those with asthma, should be a priority. Launching targeted campaigns to inform parents about the dangers of vaping and its specific implications for children with asthma should be strengthened. Studies on the association between timing, exposure patterns, asthma development, exacerbations, and the long-term effects of vaping, especially in asthmatic children and adolescents, are urgently needed to carry out targeted and effective preventive interventions. While our findings must be confirmed by future longitudinal studies, considering screening for and documenting SHE to ENDS aerosols among children and adolescents with asthma might be beneficial for health professionals. Incorporating ENDS aerosol exposure as a potential trigger in asthma self-management/action plans and updating asthma action plans to include specific guidelines for addressing vaping-related issues should be contemplated, as well as appropriate legislation that restricts access to vaping products for minors, enforces penalties for those who provide e-cigarettes to children, and implements measures to stop the marketing of vaping products among children and adolescents.

## Figures and Tables

**Table 1:** Demographic and clinical findings of the enrolled population (*n* = 54).

	Group A (Exposed)	Group B (Unexposed)
**Age, mean ± SD, years**	9.7 ± 3.6	11.8 ± 4.1
**Gender, *n* (%)**	Male, 16 (59.3) Female, 11 (40.7)	Male, 16 (59.3) Female, 11 (40.7)
**Race, *n* (%)**	Caucasian, 26 (96.3) Asian, 1 (3.7)	Caucasian, 25 (92.6) Asian, 1 (3.7) African, 1 (3.7)
**Home location, *n* (%)**	City, 17 (63) Countryside, 10 (37)	City, 15 (55.6) Countryside, 12 (44.4)
**Atopic comorbidities, *n* (%)**	AR, 18 (66.7)	AR, 22 (81.5) FA, 2 (7.4)
**Treatment with omalizumab, *n* (%)**	1 (3.7)	1 (3.7)

(AR: allergic rhinitis; FA: food allergy; *n*: number; SD: standard deviations).

**Table 2:** Result of the statistical analysis.

Second-Hand Exposure to E-Cigarettes	Group A (Yes)	Group B (No)	*p*
Exacerbations, mean ± SD, *n*/year	7.7 ± 6.8	5.7 ± 6.1	0.27
Need for rescue therapy, *n* (%)	6	1	0.19
Need for therapeutic step-up, *n* (%)	44.4	29.6	0.3
ACT/c-ACT score, mean ± SD	17.2 ± 7.6	19.6 ± 3.8	0.3
	18.3 ± 5.6	14.6 ± 5.8	0.4

(ACT: Asthma Control Test; c-ACT: children-Asthma Control Test; *n*: number; SD: standard deviations).

## Data Availability

The data presented in this study are available on request from the corresponding author.
